# Dietary Patterns of European Children and Their Parents in Association with Family Food Environment: Results from the I.Family Study

**DOI:** 10.3390/nu9020126

**Published:** 2017-02-10

**Authors:** Antje Hebestreit, Timm Intemann, Alfonso Siani, Stefaan De Henauw, Gabriele Eiben, Yiannis A. Kourides, Eva Kovacs, Luis A. Moreno, Toomas Veidebaum, Vittorio Krogh, Valeria Pala, Leonie H. Bogl, Monica Hunsberger, Claudia Börnhorst, Iris Pigeot

**Affiliations:** 1 Leibniz-Institute for Prevention Research and Epidemiology—BIPS, 28359 Bremen, Germany; intemann@leibniz-bips.de (T.I.); leonie-helen.bogl@helsinki.fi (L.H.B.); boern@leibniz-bips.de (C.B.); pigeot@leibniz-bips.de (I.P.); 2 Institute of Food Sciences, National Research Council, 83100 Avellino, Italy; asiani@isa.cnr.it; 3 Department of Public Health, Ghent University, 9000 Ghent, Belgium; stefaan.dehenauw@ugent.be; 4 Department of Public Health and Community Medicine, University of Gothenburg, 40530 Gothenburg, Sweden; gabriele.eiben@medfak.gu.se (G.E.); monica.hunsberger@gu.se (M.H.); 5 Research and Education Institute of Child Health, 2035 Strovolos, Cyprus; kourides@cytanet.com.cy; 6 Institute for Medical Information Processing, Biometrics and Epidemiology, Ludwig-Maximilians-Universität München, 81377 Munich, Germany; eva.kovacs@med.uni-muenchen.de; 7 German Center for Vertigo and Balance Disorders, Ludwig-Maximilians-Universität München, 81377 Munich, Germany; 8 GENUD (Growth, Exercise, Nutrition and Development) Research Group, Instituto Agroalimentario de Aragón (IA2), Instituto de Investigación Sanitaria Aragón (IIS Aragón), Centro de Investigación Biomédica en Red Fisiopatología de la Obesidad y Nutrición (CIBERObn), University of Zaragoza, 50009 Zaragoza, Spain; lmoreno@unizar.es; 9 Department of Chronic Diseases, National Institute for Health Development, 11619 Tallinn, Estonia; toomas.veidebaum@tai.ee; 10 Department of Preventive and Predictive Medicine, Fondazione IRCCS Istituto Nazionale dei Tumori, 20133 Milan, Italy; Vittorio.Krogh@istitutotumori.mi.it (V.K.); Valeria.Pala@istitutotumori.mi.it (V.P.); 11 Department of Public Health, University of Helsinki, 00014 Helsinki, Finland; 12 Finnish Institute of Molecular Medicine, University of Helsinki, 00014 Helsinki, Finland

**Keywords:** food consumption, family resemblance, cluster analysis, shared meals, soft drink, childhood obesity

## Abstract

The aim of this study was to determine whether an association exists between children’s and parental dietary patterns (DP), and whether the number of shared meals or soft drink availability during meals strengthens this association. In 2013/2014 the I.Family study cross-sectionally assessed the dietary intakes of families from eight European countries using 24-h dietary recalls. Usual energy and food intakes from six- to 16-year-old children and their parents were estimated based on the NCI Method. A total of 1662 child–mother and 789 child–father dyads were included; DP were derived using cluster analysis. We investigated the association between children’s and parental DP and whether the number of shared meals or soft drink availability moderated this association using mixed effects logistic regression models. Three DP comparable in children and parents were obtained: Sweet & Fat, Refined Cereals, and Animal Products. Children were more likely to be allocated to the Sweet & Fat DP when their fathers were allocated to the Sweet & Fat DP and when they shared at least one meal per day (OR 3.18; 95% CI 1.84; 5.47). Being allocated to the Sweet & Fat DP increased when the mother or the father was allocated to the Sweet & Fat DP and when soft drinks were available (OR 2.78; 95% CI 1.80; 4.28 or OR 4.26; 95% CI 2.16; 8.41, respectively). Availability of soft drinks and negative parental role modeling are important predictors of children’s dietary patterns.

## 1. Introduction

Family members share similar eating habits that are affected by individual factors and the family food environment [1]. Parental role modeling and perception of adequacy of their child’s diet are important predictors for the child’s current dietary behavior [2] and watching the parents eat raises the children’s awareness of their parents’ eating behaviors [3,4]. Despite the fact that fathers and mothers were found to influence the child’s eating behavior [5,6], the influence differs for mothers compared to fathers [7,8]. Paternal dietary influence was identified for fruit but also for fat-and energy-dense, nutrient-poor foods [9,10], whereas positive child–mother correlations have been reported for fruit and vegetable intake [11] and soft drinks [12]. Thus, parents build their children’s food environment by making healthy foods [13] or unhealthy foods [14] available. Accordingly the children’s food consumption was associated with healthy foods (so-called core foods, e.g., cereals, dairy, fruit, and vegetables) or with unhealthy non-core foods (e.g., snack foods, fats, and oils) [5]. As an example, adolescents were more likely to consume fruit and vegetables when parents made those foods available [15,16]. It has been observed that the person who prepares the majority of family meals largely influences the consumption of fruit and vegetables but also high-fat foods; this association increases with increasing numbers of shared meals [17]. 

Previous research has demonstrated that the association between parental and child intake increased with an increasing number of family meals at home [18] and that the number of family meals was positively associated with the consumption of healthier foods [19]. Family mealtimes provide structure and a regular opportunity for developing emotional connections among family members and therefore help children to monitor their mood and learn healthy dietary behaviors [20]. Accordingly, higher family meal frequency was found to be associated with significantly fewer weekly servings of sweets and sugar-sweetened beverages [21]; however, the consumption of those non-core foods (e.g., sugar sweetened beverages) was found to be higher when their home availability was higher [5]. Consumption of sugar-sweetened beverages is one epidemiological key health indicator of the European Core Health Indicators [22] and is frequently used in public health monitoring, especially when addressing socioeconomic determinants of eating behavior in European children and adolescents [23,24]. Investigations in low-income parent–child dyads found that soft drink availability at home was a strong influencing factor for the children’s soft drink intake [25], identifying parents as gatekeepers for the family food environment. 

Apart from this literature, it is striking that there is little knowledge about the resemblance of entire dietary patterns among children and their parents across Europe, which was described in the present study. The previous literature mainly investigated parental influence on the children’s intake of particular food groups such as fruit and vegetables or sugar-sweetened beverages. We therefore aimed at adding knowledge on the influence of the entire parental DP on the children’s DP. Besides parental intake, home availability has also been found to predict children’s intake of core-food and non-core foods. Thus, we aimed at determining whether the family food environment (operationalized as the number of shared meals and availability of soft drinks during meals) moderated the association between children’s DP and parental DP. Understanding to what extent the family food environment, along with the parental DP, influences children’s eating behavior has important public health implications, because in this age children and adolescents mostly still live with their parents and potentially eat up to three meals a day at home. Development of intervention strategies to improve children’s dietary patterns is likely to be more successful if supported by an understanding not only healthy but also unhealthy food intake.

## 2. Materials and Methods

### 2.1. Study Participants

Data from this investigation were obtained from the I.Family cohort. In 2013/2014 the I.Family study cross-sectionally examined children and parents from Sweden, Germany, Hungary, Italy, Cyprus, Spain, Belgium, and Estonia in order to investigate associations between eating habits and lifestyle factors leading to overweight and obesity [26]. For this investigation children and adolescents from six years to approximately 16 years who lived with their families were invited to the examination, together with the person having the care and custody of the child (hereinafter named parents). In the present analysis we included children and parents providing at least one 24HDR (*N* = 4816). In the final mixed effects logistic regression model, we included 1662 child–mother dyads (with 1269 mothers) and 789 child–father dyads (with 566 fathers); of those, 516 families provided information from siblings and 362 families provided information from the mother and father. Information on the availability of soft drinks during meals was provided for 1607 child–mother dyads and 763 child–father dyads.

Parents and children older than 16 years provided written informed consent. Younger children gave oral consent for examinations and sample collection. Study subjects and their parents could consent to single components of the study while abstaining from others. Study participants did not undergo any procedures unless they (and their parents) had given consent for examinations, collection of samples, subsequent analysis, and storage of personal data and collected samples. All applicable institutional and governmental regulations concerning the ethical use of human volunteers were followed during this research. Each participating center obtained ethical approval from the local responsible authorities in accordance with the ethical standards of the 1964 Declaration of Helsinki and its later amendments. 

### 2.2. Questionnaires and Anthropometric Measurements

Questionnaires were developed in English, translated into local languages, and then back-translated to check for translation errors. Parents reported the age and sex of their children and themselves in addition to their highest educational level according to the International Standard Classification of Education (ISCED) [27], which was used as a proxy indicator for the socioeconomic status (SES) of the family. Additionally, parents reported if soft drinks are available at home during meals (answer options: Yes, often or always; No or rarely). 

The field methods comprised anthropometric measurements of standing height (cm) using a Seca 225 stadiometer (Seca GmbH & KG, Birmingham, UK) in accordance with international standards for anthropometric assessment and weight (kg) [28]. Body weight was assessed in fasting status using a prototype of the TANITA BC 420 SMA digital scale for children and a TANITA BC 418 MA for adolescents and adults (TANITA Europe GmbH, Sindelfingen, Germany). All measurements were performed in light clothing (e.g., underwear) [29]. 

The BMI of the participants was calculated by dividing body weight in kilograms by squared body height in meters. The BMI of children was transformed to an age- and sex-specific *z*-score according to Cole et al. [30]. Weight groups (thin/normal and overweight/obese) of children were categorized using age- and sex-specific cutoff values based on the extended IOTF criteria [31]. Weight groups of adolescents and parents above 18 years were calculated using WHO cutoffs [32]. Even though weight status was not a focus of this investigation, it was calculated for a better characterization of the study population.

### 2.3. Dietary Information

Dietary intake of the previous 24 h was assessed using an online 24-h dietary recall (24HDR) assessment program, called ‘Self-Administered Children, Adolescents and Adult Nutrition Assessment’ (SACANA), based on the validated SACINA offline version [33]. The instrument has been validated and results supported the validity of SACANA as a self-reporting instrument for assessing intakes in children (publications in progress). 

Children and parents were asked to recall their diet and to enter the type and amount (g) of all drinks and foods consumed during the previous day, starting with the first intake after waking up in the morning. Children under 11 years were advised to ask their parents for help [34]. Study participants above 11 years of age could ask for assistance from a dietician or trained study nurse during the survey examinations, but the majority of participants had no questions since they already participated in the IDEFICS study and were therefore familiar with the recall procedures and software structure used. Standardized photographs were used to assist with accurate estimation of portion size [35]. In the present study, participants were asked to complete at least three 24HDR during the upcoming four weeks. However, the availability of repeated 24HDR varied among individuals from one to four recalls. For 43% of parents (39% of children), three repeated 24HDR were available.

The total number of main meals (breakfast, lunch, and dinner) per participant was calculated. Breakfast was defined as “shared” if the total number of shared breakfasts (parent with child) divided by the number of all reported breakfasts of the respective parent was at least 0.5. Shared lunches and dinners were categorized accordingly. The sum of all shared main meals per parent was calculated and the following categories were derived: (1) <1 shared meal per day and (2) ≥1 shared meal per day.

### 2.4. Dietary Data Analysis

Missing or implausible values for intake of single food items that could not be corrected were imputed by country, food group, and age-specific median intakes (0.15% of the entries). Incomplete 24HDR (recalls that have not been completed throughout) and those with more than four imputed values were excluded from the analysis. 

Age- and sex-specific Goldberg cutoffs were applied to classify each recall day as under-reported, plausibly reported, and over-reported energy intake, as described elsewhere [36].

In total, we excluded 955 participants classified as misreporters from the analysis: 484 children and 471 adults; among those 95% and 99% were under-reporters, and 5% and 1% were over-reporters, respectively. 

Each food recorded by SACANA was assigned to one of 15 dietary categories: healthy and unhealthy cereals and cereal products, unhealthy sugar and sugar products, healthy and unhealthy fat and fat savory sauces, healthy fruit and vegetables, healthy and unhealthy meat and meat products, healthy meat alternatives, healthy and unhealthy milk and dairy products, healthy and unhealthy non-alcoholic beverages, healthy and unhealthy mixed dishes (Table 1). Foods were categorized as “healthy” when they contained less energy, less sugar, less (unhealthy) fat, or more fiber than the unhealthy food alternative, e.g., table water (healthy beverage) vs. juice (unhealthy beverage), plain yogurt (healthy) vs. full fat and sweetened yogurt (unhealthy). Consumption of unhealthy mixed dishes was so rare that this category was not included in further analysis. 

After food categorization, individual usual daily energy intake (EI, kcal/day) and individual usual intakes of dietary categories (kcal/day, healthy non-alcoholic beverages: g/day) were estimated based on the U.S. National Cancer Institute Method [37,38]. This method allows the inclusion of covariates like age and additional food frequency information, accounts for different intake on weekend vs. work days, and corrects for the variance inflation caused by the daily variation in diet. Usual intakes were estimated for children as well as for their parents, stratified by sex (all participants with at least one plausible 24HDR). Age was considered as a covariate in all models. When estimating usual food intakes, the corresponding food consumption frequency obtained from the I.Family food frequency questionnaire was also used as a covariate to improve estimates (except for mixed dishes, as this food group was not queried in the food frequency questionnaire but was a generic category in SACANA food groups). The I.Family food frequency questionnaire was built on the valid and reproducible IDEFICS study food frequency questionnaire, which was described in detail previously [39,40,41]. The FFQ contained 59 food items comparable to those in the SACANA web tool, thus allowing categorizing the food items according to the 15 dietary categories mentioned above. The answer possibilities in the FFQ were “never/less than once a week”, “1–3 times a week”, “4–6 times a week”, “1 time/day”, “2 times a day”, “3 times a day”, and “I have no idea”. All participants were asked to complete one FFQ for the four weeks prior to the survey examination.

The individual percentage of energy contribution from all dietary categories was calculated to correct for individual total EI. For children and adults separately, these percentages were transformed into *z*-scores using sample means and sample standard deviations. The *z*-score represents the distance between the percentage of energy contribution and the corresponding population mean in units of the standard deviation. This procedure was not applied for usual EI and usual intake of non-alcoholic beverages (g/day) since EI correction is neither reasonable for EI itself nor for the calorie-free dietary category. Therefore, age-dependent *z*-scores were derived for these variables with the Generalized Additive Models for Location, Scale, and Shape (this procedure is described in detail in Appendix A).

### 2.5. Statistical Analysis

**Figure 1:**
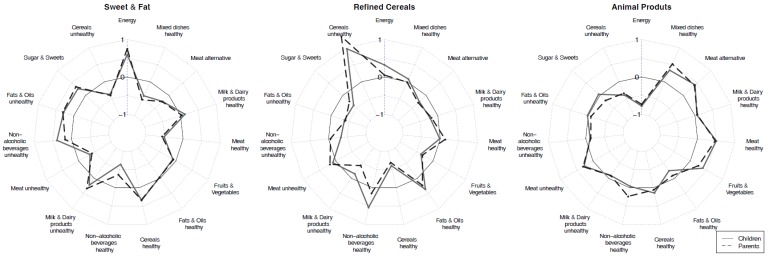
Dietary patterns of children and their parents: Sweet and Fat, Refined Cereals, and Animal Products. Mean *z*-scores of percentage of energy intake of different food groups in the three clusters for children and parents are shown (for details, see Table 2). The scale ranges from −1 to 1 with tick lines for −0.5, 0 (solid line) and 0.5.

K-means clustering was applied for children and parents separately to identify distinct clusters of participants with similar dietary patterns. In this procedure the previously derived *z*-scores were taken into account. Details of this procedure are described in Appendix A. As clusters were comparable between children and parents, the same cluster names were used. Three clusters representing the DP (Figure 1) were obtained: Sweet and Fat cluster, Refined Cereals cluster, and Animal Products cluster. Each participant was allocated to exactly one DP and corresponding indicator variables were derived (participant is in the respective cluster versus participant is not in the respective cluster).

Family food environment was operationalized using the number of shared meals (<1 or ≥1 shared meal per day) as an indicator of parental role modeling and the availability of soft drinks during meals as an indicator of home food availability. As a first step, we investigated associations between the outcome children’s DP (indicator variable) and the exposure parental DP (indicator variable) and the number of shared meals using mixed effects logistic regression. To examine whether the number of shared meals strengthened the associations between parental and children’s DP, an interaction term was included (number of shared meal × parental DP). For each of the three DP, we conducted a regression analysis separately for fathers and mothers (six models). Accordingly, we investigated in a second step the associations between children’s DP and the exposure parental DP and availability of soft drinks during meals. To examine whether the availability of soft drinks during meals strengthened the associations between parental DP and children’s DP, an interaction term (availability of soft drinks during meals × parental DP) was included. The models were adjusted for sex, age and BMI *z*-score of the children, ISCED, country, and BMI of the respective parent. In order to account for dependencies between siblings, a random effect was added for family membership. Based on the mixed effects logistic regression models, odds ratios (OR) and confidence intervals (95%CI) were calculated for a child being allocated to the DP corresponding to the parental DP depending on the number of shared meals and availability of soft drinks during meals. The analysis was performed using the procedure PROC GLIMMIX of the statistical software SAS (version 9.3; SAS Institute, Cary, NC, USA).

## 3. Results

The aims of the present study were to investigate the resemblance of children’s DP and their parents’ DP as well as to determine whether structural conditions of the family food environment moderated the association between children’s DP and parental DP.

### 3.1. Dietary Clusters

Based on dietary categories (Table 1) and usual EI, the three-cluster solutions were derived. For comparable clusters of children and parents, the following labels were assigned: Sweet and Fat (*N* = 697 for children and *N* = 728 for parents), Refined Cereals (*N* = 563 for children and *N* = 410 for parents), and Animal Products (*N* = 716 for children and *N* = 747 for parents). 

Table 2 presents the mean *z*-scores and standard deviations of usual intake for all dietary categories in the three clusters for children and parents. 

In general, we observed a resemblance of children’s dietary patterns to parents’ dietary pattern; details of these analyses can be found in Appendix A. The overall agreement between cluster allocation of children and mothers was 52% (for fathers, 53%). 

In particular, we observed the following characteristics for the three clusters representing dietary patterns (DP).

Sweet and Fat: Children and adults allocated to this cluster reported higher-than-mean intake of sugar and sweets (children’s mean 0.27; parents’ mean 0.34), unhealthy fats and oils (children’s mean 0.29; parents’ mean 0.31), unhealthy (sweetened) non-alcoholic beverages (children’s mean 0.39; parents’ mean 0.17) and unhealthy milk and dairy products (children’s mean 0.22; parents’ mean 0.34) (Figure 1). Cereals were categorized as healthy in case of low sugar content and low fat content and high fiber content (Table 1), such as whole-grain breads, plain breakfast cereals, or crispbread (children’s mean 0.34; parents’ mean 0.33). Family members allocated to this DP reported the highest EI (children’s mean 0.60; parents’ mean 0.75). 

Refined Cereals: Children and parents from this cluster reported higher-than-mean intake of unhealthy cereals (e.g., white breads, refined and/or sugared breakfast cereals, pasta from refined wheat, refined rice, sweet and/or fatty bakery products (biscuits, cakes, fritters, etc.; children’s mean 0.96; parents’ mean 1.32) and healthy fats and oils (children’s mean 0.36; parents’ mean 0.23). Both children and parents also consumed more healthy non-alcoholic beverages (children’s mean 0.54; parents’ mean 0.15). 

Animal Products: Children and parents who were allocated to this cluster reported higher intake of all types of meat (children’s mean for meat unhealthy 0.27, meat healthy 0.51; parents’ mean 0.30 and 0.48, respectively) and meat alternatives (children’s mean 0.42; parents’ mean 0.40) as well as of healthy mixed dishes (children’s mean 0.34; parents’ mean 0.52). Healthy mixed dishes were mainly based on cereals, legumes, and vegetables/potatoes, with small proportions of fish, egg, or dairy. Children and parents further reported higher-than-mean intakes of fruit and vegetables (children’s mean 0.39; parents’ mean 0.25). Parents reported a higher-than-mean intake for healthy non-alcoholic beverages (mean 0.23). The energy intake of children and parents was lowest in this DP (children’s mean −0.78; parents’ mean −0.73). 

### 3.2. Participant Characteristics

The largest proportion of children (36%) and parents (40%) was allocated to the Animal Products cluster (Table 3). The mean age of children (11.4 years) and parents (44.2 years) was highest in the Refined Cereals cluster. Girls (39%) and mothers (44%) were mainly found in the Sweet and Fat cluster, whereas most boys and men were found in the Animal Products cluster (38% and 44%, respectively).

Most normal weight children were allocated to the Animal Products cluster (38%), whereas most overweight (39%) and obese (56%) children were allocated to the Refined Cereals cluster. Most normal weight adults (43%) were in the Sweet and Fat cluster; most overweight (41%) and obese (38%) adults were found in the Animal Products cluster. 

Children and adults from low SES families mainly belonged to the Refined Cereals cluster (76% and 71%, respectively). Children from high SES families were equally allocated to the Sweet and Fat and the Animal Products clusters (both 39%). 

In all countries—except Belgium—those cluster memberships with the highest proportion of children and parents were comparable. In Italy and Hungary most children and parents shared the Refined Cereals cluster; in Estonia, Sweden and Germany most shared the Sweet and Fat cluster; and in Cyprus and Spain most shared the Animal Products cluster. In Belgium 47% of children were found in the Animal Products cluster, but 55% of their parents in the Sweet and Fat cluster. 

### 3.3. Family Food Environment

Shared meals: Resemblance was observed between parental DP and children’s DP: the chance of the child being allocated to the Sweet and Fat DP, the Refined Cereals DP, and the Animal Products DP is higher if the mother was allocated to the same DP, independently of the number of shared meals, compared to the chance if the mother was in a different DP (Table 4). Overall, children were more likely to be allocated to the Sweet and Fat DP if the father was allocated to the same DP; the odds ratio increased with an increase in the number of shared meals from <1 to ≥1 (OR 2.30; 95% CI 1.15; 4.57 or OR 3.18; 95% CI 1.84; 5.47, respectively).

Soft drink availability during meals: The child was more likely to be allocated to the Sweet and Fat DP if soft drinks were available during meals, even if the mother was not allocated to the Sweet and Fat DP (OR 1.97; 95% CI 1.20; 3.25, Table 5). The chance of being allocated to the Sweet and Fat DP was highest if the mother was allocated to the Sweet and Fat DP and soft drinks were available during meals (OR 2.78; 95% CI 1.80; 4.28). The child was more likely to be allocated to the Refined Cereals DP or the Animal Products DP if the mother was allocated to the same DPs and if no soft drinks were available during meals (OR 2.48; 95% CI 1.43 and 4.27; OR 2.16;1.59; 2.92, respectively). The child was most likely to be allocated to the Sweet and Fat DP, the Refined Cereals DP, and the Animal Products DP if the father was allocated to the respective DP and if soft drinks were not available during meals (OR 2.48; 95% CI 1.58; 3.87, OR 2.05; 95% CI 1.22; 3.45 and OR 2.48; 95% CI 1.62; 3.79, respectively). The chance of the child sharing the father’s Sweet and Fat DP is higher if soft drinks are available during meals (OR 4.26; 95% CI 2.16; 8.41).

## 4. Discussion

The present study suggests important similarities between children’s and parental DP. Three DP were obtained in this multi-country study: Animal Products, Refined Cereals, and Sweet and Fat. To our knowledge, this is the first study presenting the resemblance of the DP of pan-European children and their parents using cluster analysis. The study was further able to describe how the family food environment (operationalized as the number of shared meals and the availability of soft drinks during meals) moderated the association between children’s DP and parental DP. 

Resemblance of dietary patterns between children and parents: Previously, maternal consumption of core foods (e.g., cereals, dairy, fruit, and vegetables) and non-core foods (e.g., snack foods, fats, and oils) has been shown to be associated with a child’s higher intake of the same foods [5]. Mothers tend to be the person habitually preparing the family meals [17] and mothers reported greater perceived responsibility for feeding their children [18]. Women are known to exert positive influence on children’s food consumption [42] because they are more likely to adhere to dietary guidelines [43]. This is in line with our findings that identified the influencing nature of the maternal Animal Products DP per se and when the mother was eating with the child: in our study the Animal Products DP was characterized through the above-the-mean intake of healthy food alternatives such as fruit vegetables, healthy alternatives for meat, meat substitutes, milk and dairy products, cereals, and mixed dishes (Figure 1). Reported EIs were lowest in the Animal Products DP and we observed the highest proportion of normal weight children in this DP but the highest proportion of overweight parents. Fathers’ influence on the child’s food choices was highest for the foods of the Sweet and Fat DP including all types of sugar and sweets, unhealthy fats and oils, unhealthy beverages, and unhealthy milk and dairy products. In particular, the *z*-scores for non-alcoholic unhealthy beverages (including also soft drinks) were highest in the Sweet and Fat DP compared to the other two DP. Likewise, previous studies have reported that fathers have primary influence on the children’s intake of non-core foods [8]. 

Dietary patterns and shared meals: In our study associations were found between children’s DP and maternal DP independently of the number of shared meals and in particular between children’s DP and fathers’ Sweet and Fat DP if ≥1 meal was eaten together. Also in previous studies, paternal influence has been found to predict child’s food intake in that fathers used pressure tactics whereas mothers praised children for eating certain things [44]. In particular, Robinson et al. [7] observed strong correlations for foods typically eaten at breakfast such as grains and fruit in child–father dyads for families with working mothers, indicating that fathers have breakfast with their children when mothers leave home early. Children and adolescents sharing three or more meals per week with the family had healthier dietary patterns compared to those who share fewer than three family meals [45]. This is in contrast to our findings, where children who eat together with their fathers at least once a day were more likely to share the Sweet and Fat DP with their fathers than sharing the generally healthier Animal Products DP. 

Dietary patterns and the availability of soft drinks during meals: Although the mechanisms for how family meals facilitate healthy eating behaviors have not been empirically explained, different approaches are currently discussed. Eating together is an important ritual for interacting with family members and offers opportunities for children to learn about eating by watching others [3]. Also, low availability and consumption of convenient foods or sodas during family meals can contribute to healthy dietary intake patterns [46]. On the other hand, the availability of soft drinks during shared meals and parental soft drink consumption were associated with the child’s soft drink consumption (Sweet and Fat DP) in our study. This is in line with earlier findings from U.S. studies where parental food choices [3] and soft drink availability were strong influencing factors for the children’s intake [25], identifying parents as gatekeepers for the family food environment and as role models. Those foods (preferred and) consumed by the parents were the foods to which children were routinely exposed and shaped the children’s food preferences and consumption [47,48]. It is not surprising that the availability of soft drinks and chips has been observed to be greater in families who frequently consumed fast food during family meals [49]. We therefore suggest that home availability of foods is an important predictor for children’s preferences, even more so if parents choose the same foods during meals [50]. Making healthy foods available and also eating those foods may enhance children’s understanding and acceptance of a healthy diet [51].

### Limitations and Strengths

In the I.Family study dietary information was mainly given by self-respondents. Self-reporting can be susceptible to reporting bias [52]. We therefore followed a rigorous approach in order to reduce errors due to portion size estimation, incomplete recalls, misreporting, or daily variations in intake. Firstly, the development of the SACANA computer-assisted assessment tool with standardized photographs, multiple plausibility checks, and reminding questions facilitated the reporting of accurate portion sizes and complete recall. Secondly, as a first step in the data analysis, the exclusion of incomplete recalls and recalls with implausible energy reporting helped to correct for reporting bias. Individuals with misreported EI (under-reporters: 462 children, 465 adults; over-reporters: 22 children, six adults) were more likely to be female, from medium educated families, and from Estonia or Germany. They were more often overweight and obese (66% of adults; 36% of children) compared to plausible reporters (16% adults, 4% children). In a separate analysis we derived the clusters including the 955 misreporters in order to compare cluster memberships of the plausible reporters sample (1662 child–mother dyads and 789 child–father dyads) with the cluster memberships of the full study sample (2269 child–mother dyads and 1058 child–father dyads). After comparing the cluster membership of the full sample (also including misreporters) with the final study sample (plausible reporters only) we observed that the three DP remained comparable, except that EI was found to be lower in the Animal Products cluster when misreporters were included. As the Animal Products cluster included 58% of misreporting children and 83% of misreporting parents, including misreporters would overestimate intake (particularly in the Animal Product cluster) and underestimate the intake of plausible reporters (particularly in the Refined Cereals and Sweet and Fat clusters), given that we consider the percentage of energy contribution from dietary categories. We therefore decided to exclude the misreporters from the analysis. However, the question of how to handle possibly implausible interviews has not been answered conclusively: the inclusion of misreports may obscure or even inverse diet–disease relationships, as recently reported, and adjustment for the reporting group may also lead to bias [53]. Finally, deriving the usual intake based on the NCI method [37] and accounting for day-to-day variation in intake is a clear strength of this study. 

As might be expected, we observed a resemblance between the parental DP and children’s DP. These improved relations do not reflect reporting bias (due to proxy-reporting of dietary intake), as reported in other studies [18]. In the present study, participants personally reported dietary intake; thus the strength of association between child–mother dyads and child–father dyads truly reflect the environmental influence of parents on the child’s DP and can be seen as an additional strength of this study. In general, the I.Family study allows a deep insight into the resemblance of DP among family members across Europe and the influence of parental DP on their child’s DP when eating together or not. The large sample size comprises data from eight European countries; the strictly standardized data assessment, documentation, and data cleaning processing guarantee the highest possible data quality. 

## 5. Conclusions

Using cluster analysis to derive dietary patterns allowed us to compare groups of European children and their parents with different dietary profiles and to examine the effect of daily number of shared meals and soft drink availability during meals on the association between child’s and parental DP. The availability of soft drinks during meals and negative parental role modeling are important predictors for intake of sweet and fat foods in children. Intervention strategies should focus on healthy shopping choices. Parents as gatekeepers for home food availability and as role models for children’s eating behavior should be counseled in which foods should be consumed on a regular basis and which foods should be avoided at home. Not purchasing unhealthy foods will decrease their availability at home and during meals; thus, their consumption may be hampered and may decline in parents and their children.

## Figures and Tables

**Table 1:** Food groups and healthy/unhealthy dietary categories.

Food Group	Healthy Alternative	Unhealthy Alternative
Cereals & cereal products	Low sugar content (<15%), low fat content (<20%) and high fiber content (≥5%)	≥15% sugar, ≥20% fat, and <5% fiber content
Sugar & sweets	-	Sugar, sweets, candies, marzipan, chocolate, nut spreads, jam, honey, ice cream, canned/sugared fruit, etc.
Fats & oils	From mainly plant origin, and for sauces <40% fat content	Mainly animal and processed origin and ≥40% fat for sauces
Non-alcoholic beverages	Non-caloric and non-processed beverages such as table water, plain herbal teas, plain coffee	Sweetened and processed beverages: manufactured juices, sodas, ice tea, energy drinks, coffee/tea with milk/sugar, broth
Meat	Containing <10% fat, and meat products with <20% fat from poultry, rabbit or game	Meat from all other origins than poultry, including offal, with ≥10% fat and meat products containing ≥20% fat
Meat alternative	Soy products, meat and dairy substitutes from soy, vegetarian burgers, tempeh, tofu, seitan and lean prepared fish and eggs	-
Milk & dairy products	Low fat and unsweetened	Full fat and sweetened, flavored
Fruit & vegetables	Fresh fruit and vegetables, their fresh juices/smoothies, or lean preparation, without added sugars	-
Mixed dishes	Based on cereals, legumes, vegetables/potatoes with small amounts of fish, egg or dairy, soups, veloutés, mixed salad	Based on meat; fried foods (also fried vegetables), fast food, snack foods (not included in the final cluster analysis)

**Table 2:** *z*-scores of usual intake in the three clusters for children and for parents (mean values and standard deviations).

**Children**	**Sweet & Fat (*N* = 697; 35%)**		**Ref. Cereals (*N* = 563; 28%)**		**Animal Products (*N* = 716; 36%)**	
	**Mean**	**SD**	**Mean**	**SD**	**Mean**	**SD**
Energy	0.60 ^a^	0.82	0.32	0.83	−0.78 ^b^	0.79
Non-alcoholic beverages—healthy	−0.64 ^b^	0.89	0.54 ^a^	0.81	−0.04	1.01
Non-alcoholic beverages—unhealthy	0.39 ^a^	1.24	−0.33 ^b^	0.67	−0.12	0.82
Cereals—unhealthy	−0.37	0.7	0.96 ^a^	1.06	−0.40 ^b^	0.61
Cereals—healthy	0.34 ^a^	1.05	−0.61 ^b^	0.64	0.14	0.97
Sugar & Sweets	0.27 ^a^	0.94	−0.39 ^b^	0.94	0.05	1.00
Fats & Oils—unhealthy	0.29 ^a^	1.21	−0.38 ^b^	0.61	0.02	0.93
Fats & Oils—healthy	−0.03	1.03	0.36 ^a^	1.18	−0.25 ^b^	0.69
Fruit & Vegetables	−0.09	0.96	−0.37 ^b^	0.87	0.39 ^a^	1.00
Meat—unhealthy	−0.35 ^b^	0.88	0.09	1.02	0.27 ^a^	1.00
Meat—healthy	−0.51 ^b^	0.73	−0.01	0.96	0.51 ^a^	1.01
Milk & Dairy products—unhealthy	0.22 ^a^	1.13	−0.15 ^b^	0.91	−0.1	0.89
Milk & Dairy products—healthy	0.12 ^a^	1.09	−0.22 ^b^	0.82	0.05	1.02
Meat alternative	−0.24	0.78	−0.25 ^b^	0.71	0.42 ^a^	1.22
Mixed dishes—healthy	−0.41 ^b^	0.89	0.08	0.97	0.34 ^a^	0.98
**Parents**	**Sweet & Fat (*N* = 728; 39%)**		**Ref. Cereals (*N* = 410; 22%)**		**Animal Products (*N* = 747; 40%)**	
	**Mean**	**SD**	**Mean**	**SD**	**Mean**	**SD**
Energy	0.75 ^a^	0.76	0.05	0.83	−0.73 ^b^	0.75
Non-alcoholic beverages—healthy	−0.36 ^b^	1.01	0.15	0.87	0.23 ^a^	0.96
Non-alcoholic beverages—unhealthy	0.17 ^a^	1.12	−0.05	1.03	−0.14 ^b^	0.82
Cereals—unhealthy	−0.40 ^b^	0.59	1.32	1.09	−0.34	0.55
Cereals—healthy	0.33 ^a^	1.02	−0.69 ^b^	0.58	0.06	0.97
Sugar & Sweets	0.34 ^a^	0.96	−0.24 ^b^	0.93	−0.20	0.99
Fats & Oils—unhealthy	0.31 ^a^	1.17	−0.41 ^b^	0.66	−0.08	0.87
Fats & Oils—healthy	−0.03	1.04	0.23 ^a^	1.11	−0.09 ^b^	0.87
Fruit & Vegetables	−0.08	0.94	−0.32 ^b^	0.95	0.25 ^a^	1.02
Meat—unhealthy	−0.41 ^b^	0.87	0.18	0.91	0.30 ^a^	1.03
Meat—healthy	−0.56 ^b^	0.74	0.13	0.84	0.48 ^a^	1.03
Milk & Dairy products—unhealthy	0.34 ^a^	1.14	−0.42 ^b^	0.72	−0.09	0.87
Milk & Dairy products—healthy	0.03	1.02	−0.18 ^b^	0.93	0.07 ^a^	1.00
Meat alternative	−0.25	0.77	−0.29 ^b^	0.65	0.40 ^a^	1.21
Mixed dishes—healthy	−0.53 ^b^	0.91	−0.01	0.84	0.52 ^a^	0.89

^a^ The highest mean value within a row; ^b^ The lowest mean value within a row.

**Table 3:** Characteristics of the study population, including plausible reporters stratified by cluster membership and misreporters (number and percentages).

Covariates	Plausible Reporters												Misreporters			
	Children, Adolescents						Parents						Children, Adolescents		Parents	
	Sweet & Fat (*N* = 697; 35%)		Ref. Cereals (*N* = 563; 28%)		Animal Products (*N* = 716; 36%)		Sweet & Fat (*N* = 728; 39%)		Ref. Cereals (*N* = 410; 22%)		Animal Products (*N* = 747; 40%)		(*N* = 484)		(*N* = 471)	
Age mean (SD)	10.9 (2.1)		11.4 (2.1)		11.2 (2.1)		41.8 (5.4)		44.2 (5.8)		41.8 (5.4)		12.2 (1.9)		42.3 (5.7)	
Age range (min; max)	6.0; 15.8		6.0; 16.0		6.0; 16.0		28.1; 58.5		30.3; 65.4		27.0; 63.0		6.5; 15.7		27.8; 58.7	
Sex (*N*, %)																
Male	322	32	311	30	387	38	149	26	173	30	257	44	238	49	139	30
Female	375	39	252	26	329	34	579	44	237	18	490	38	246	51	332	71
Weight group (*N*, %N) ^a^																
Normal weight	588	37	403	25	613	38	428	43	175	18	388	39	310	64	159	34
Overweight	93	32	115	39	84	32	209	34	157	25	255	41	123	25	156	33
Obese	16	20	45	56	19	24	91	33	78	29	104	38	51	11	156	33
ISCED-Level of parents ^b^ (*N*, %)																
Low Education	5	12	31	76	5	12	1	3	24	71	9	26	26	5	31	7
Medium Education	200	31	245	38	207	32	185	30	190	31	234	38	227	47	235	50
High Education	482	39	271	22	491	39	529	44	182	15	488	41	226	47	199	42
Missing ISCED ^c^	10	26	16	41	13	33	13	30	14	33	16	37	5	1	6	1
County (*N*, %)																
Italy	8	3	232	85	32	12	14	6	179	77	38	16	59	12	77	16
Estonia	275	50	29	5	246	45	341	56	28	5	244	40	148	31	82	17
Cyprus	28	17	66	39	75	44	33	21	40	26	81	53	27	6	41	9
Belgium	67	44	14	9	73	47	66	55	14	12	39	33	24	5	18	4
Sweden	133	39	120	35	89	26	113	40	58	20	113	40	55	11	66	14
Germany	149	49	40	13	113	37	133	47	39	14	114	40	143	30	147	31
Hungary	29	32	38	42	23	26	11	12	41	45	39	43	17	4	25	5
Spain	8	8	24	25	65	67	17	16	11	10	79	74	11	2	15	3

^a^ Weight categories according to Cole et al. [30] for children and according to WHO for adults; ^b^ International Standard Classification of Education Maximum (ISCED); maximum of both parents (0, 1, 2 = low education; 3, 4 = medium education; 5, 6 = high education); ^c^ Those individuals with missing ISCED information were excluded from mixed effects logistic regression models.

**Table 4:** Odds Ratios (OR) and confidence intervals (CI) for a child being allocated to a dietary pattern depending on parental dietary pattern and number of shared daily meals, given by sex of parents; all models were adjusted for age and BMI *z*-score of child, parental BMI, highest ISCED of family, and country of residence.

Parental Dietary Pattern	Children’s Dietary Pattern								
	Sweet & Fat			Ref. Cereals			Animal Products		
	*N*	OR	95%CI	*N*	OR	95%CI	*N*	OR	95%CI
Maternal dietary pattern (*N* = 1662)									
Different & <1 shared meal (reference)	132	1.00		265	1.00		183	1.00	
Different & ≥1 shared meals	771	0.97	0.59; 1.58	1096	1.20	0.70; 2.07	877	1.06	0.70; 1.60
Identical & <1 shared meal	158	2.12	1.18; 3.81	25	5.70	1.51; 21.48	107	2.18	1.21; 3.92
Identical & ≥1 shared meals	601	1.91	1.17; 3.13	276	2.70	1.34; 5.45	495	2.19	1.41; 3.40
Paternal dietary pattern (*N* = 789)									
Different & <1 shared meal (reference)	149	1.00		153	1.00		112	1.00	
Different & ≥1 shared meals	430	1.31	0.82; 2.09	396	0.83	0.45;1.54	338	0.55	0.32; 0.92
Identical & <1 shared meal	58	2.30	1.15; 4.57	54	1.66	0.68;4.06	95	1.45	0.78; 2.71
Identical & ≥1 shared meals	152	3.18	1.84; 5.47	186	1.99	0.98;4.08	244	1.54	0.91; 2.59

**Table 5:** Odds Ratios (OR) and confidence intervals (CI) for a child being allocated to a dietary pattern depending on parental dietary pattern and availability of soft drinks during meals, given by sex of parents; all models were adjusted for age and BMI *z*-score of child, parental BMI, highest ISCED of family, and country of residence.

Parental Dietary Pattern	Children’s Dietary Pattern								
	Sweet & Fat			Ref. Cereals			Animal Products		
	*N*	OR	95%CI	*N*	OR	95%CI	*N*	OR	95%CI
Maternal dietary pattern (*N* = 1607)									
Different & soft drink not available (reference)	742	1.00		1017	1.00		767	1.00	
Different & soft drink is available	138	1.97	1.20; 3.25	294	0.46	0.25; 0.84	256	0.95	0.65; 1.38
Identical & soft drink not available	521	2.04	1.49; 2.80	246	2.48	1.43; 4.27	496	2.16	1.59; 2.92
Identical & soft drink is available	206	2.78	1.80; 4.28	50	1.67	0.66; 4.22	88	1.42	0.82; 2.47
Paternal dietary pattern (*N* = 763)									
Different & soft drink not available (reference)	465	1.00		407	1.00		360	1.00	
Different & soft drink is available	92	1.55	0.90; 2.68	122	0.43	0.18; 1.04	80	0.83	0.45; 1.52
Identical & soft drink not available	151	2.48	1.58; 3.87	209	2.05	1.22; 3.45	256	2.48	1.62; 3.79
Identical & soft drink is available	55	4.26	2.16; 8.41	25	1.97	0.61; 6.39	67	1.80	0.96; 3.36

## Appendix A

In the appendix we give details on (1) the derivation of *z*-scores of usual energy intake and *z*-scores of healthy non-alcoholic beverages and (2) on the clustering procedure. Both were conducted with the statistical software R (version 3.1.0) [54].

### Appendix A.1. Derivation of z-Scores

In contrast to the intake of the food groups (measured in kcal/day) the usual energy intake (kcal/day) and usual intake of healthy non-alcoholic beverages (g/day) cannot be reasonably adjusted for total energy intake. Therefore, we decided to adjust energy intake and the percentage of intake contribution from healthy non-alcoholic beverages for age and sex as a surrogate for adjustment for total energy intake, since age and sex are strongly associated with total energy intake. We applied the Generalized Additive Models for Location, Scale, and Shape (GAMLSS) [55] in order to derive sex- and age-specific *z*-scores for these variables. GAMLSS allows for deriving sex- and age-specific percentile curves. Stratified by sex and separately for children and parents, such curves were estimated. By default, we used a GAMLSS model consisting of Box–Cox power exponential distribution. The distribution parameters were modeled with penalized B-splines depending on age. If this default model did not converge or provided inadequate results, we used the Box–Cox Cole and Green distribution (BCCG) and/or cubic splines instead: For energy intake in men and intake of healthy non-alcoholic beverages in women, we used the BCCG distribution; for healthy non-alcoholic beverages in boys we used cubic splines. According to the estimated curves, values of usual energy intake and intake of healthy non-alcoholic beverages were transformed into individual *z*-scores.

The interpretation of the *z*-scores derived by GAMLSS differs slightly from the common z-scores of the food groups (measured in kcal/day): For the common *z*-score, a positive (negative) value indicates a value above (below) the mean percentage of energy from the specific food group, whereas for the *z*-scores derived by GAMLSS, a positive (negative) value indicates a value above (below) the sex- and age-specific mean. However, for both types of *z*-scores, the mean is zero and the standard deviation is one.

### Appendix A.2. Clustering Procedure

Cluster analysis using the k-means approach by Hartigan and Wong [56] was applied to identify clusters of children and clusters of parents with similar dietary patterns. The usual intake variables (sex- and age-adjusted *z*-scores and energy-adjusted *z*-scores) were used in the cluster analysis to find clusters of children and adults with distinct dietary patterns.

Since the true number of clusters is unknown and k-means only converge to a local minimum, the k-means algorithm was applied with 50 random starts for each number of clusters from two to eight. Accordingly, out of these 50 solutions the one with the lowest total within cluster sum of squares was chosen. For these solutions the convergence criterion was reached in fewer than nine iterations. To decide on the appropriate number of clusters, all final two to eight cluster solutions were examined: since silhouette coefficients [57] and the total within cluster sum of squares did not give a clear indication for an appropriate number of clusters, the interpretability of the clusters was used as the decision criterion. The three-cluster solution was favored. To evaluate the reproducibility of the cluster solutions, we conducted a cluster analysis in a randomly chosen subsample containing 85% of subjects [58]. This was repeated 10 times for children and adults separately. Compared to the whole sample solutions, the percentage of agreement and the adjusted Rand Index (ARI) [59] were calculated for the three-cluster solutions, leading to a mean agreement of 97% (95%) and a mean ARI of 0.92 (0.87) for adults (children).

Since the dietary patterns of children and parents were very similar, the same cluster names were used. The Euclidian distances between cluster centroids among children and adults were calculated (Table A1), showing that the distances between same labeled clusters were lowest. Two examples illustrating the Euclidean distances between children’s and parents’ DP are given: a constant difference of 0.1 between children’s and parents’ *z*-scores leads to a distance of 0.39; a constant difference of 0.6 leads to a distance of 2.32.

**Table A1:** Cross-tabulation of Euclidean distances between the cluster means of children and parents.

Parents	Children, Adolescents Sweet & Fat Ref. Cereals Animal Products		
Sweet & Fat	0.45	2.54	2.45
Ref. Cereals	2.7	0.77	2.51
Animal Products	2.48	2.25	0.49
